# Outcomes of a Novel Learning Health System Training Model for Rehabilitation Researchers

**DOI:** 10.1002/lrh2.70127

**Published:** 2026-11-13

**Authors:** Margarite J. Whitten, Kristin Ressel, Rosa R. Baier, Ann Reddy, Linda Resnik, Janet K. Freburger

**Affiliations:** ^1^ Brown University School of Public Health Providence Rhode Island USA; ^2^ University of Pittsburgh School of Health and Rehabilitation Sciences Pittsburgh Pennsylvania USA

**Keywords:** learning health system, partnered research, rehabilitation, stakeholder engagement

## Abstract

**Introduction:**

The Learning Health Systems Rehabilitation Research Network (LeaRRn) was developed with NIH funding to build Learning Health System (LHS) capacity in rehabilitation. This manuscript describes the LeaRRn LHS Scholar program, summarizes evaluation findings from the first three Scholar cohorts, and offers recommendations for future LHS training programs.

**Methods:**

We conducted a parallel convergent mixed methods analysis of program assessments completed by 18 Scholars, 12 mentors, and nine health system partners. Descriptive statistical analyses were used to describe structured survey responses. Qualitative thematic analysis was used to identify major codes and associated themes in the data. Qualitative and quantitative findings were then triangulated to inform recommendations for future LHS research training programs.

**Results:**

The LeaRRn LHS Scholar program advanced LHS capacity for both health systems and researchers. Most Scholars addressed a health system priority topic with work that would lead to improvements in care. LeaRRn supported health system partners to develop as a LHS. Most Scholars developed in five or more of AHRQ's LHS research competencies. Barriers to Scholar work included: challenges co‐designing feasible projects with limited time and resources; engaging with complex health systems that have evolving needs and competing demands; and difficulty accessing and using health system data. Facilitators of Scholar work included: strong engagement with the health system and alignment with its priorities; and active mentors with relevant research or LHS expertise. Program successes included: scholarly products, continuing partnerships, and increased capacity and appetite for further LHS work.

**Conclusions:**

The LeaRRn LHS Scholar program evaluation yielded insights that can be used to develop or strengthen future LHS training programs. This paper provides recommendations related to: program length, support, and structure; applicant selection; health system partnerships; mentoring; training expectations; health system navigation; health system financial support; administrative program support; and peer‐learning opportunities.

## 1 Introduction

There is an urgent need to improve rehabilitation care by implementing evidence‐based interventions and de‐implementing ineffective or harmful interventions. Although numerous evidence‐based rehabilitation interventions exist, translation into routine care is slow, leaving persistent evidence‐to‐practice gaps [1, 2, 3, 4, 5, 6]. Learning health systems (LHS) approaches aim to close these gaps by generating and applying evidence in real‐time and more deliberately integrating scientific discovery with healthcare delivery. While LHS research training opportunities have been offered in the U.S. through the Agency for Healthcare Research and Quality (AHRQ) and the Department of Veterans Affairs, none had previously focused on rehabilitation care [7, 8, 9, 10, 11, 12, 13]. Moreover, rehabilitation settings often lack necessary expertise and infrastructure to function as effective LHSs, limiting progress toward data‐driven, continuous care improvement.

The Learning Health Systems Rehabilitation Research Network (LeaRRn) was established in 2020 to build Learning Health System (LHS) capacity in rehabilitation. LeaRRn's mission was to strengthen rehabilitation researchers' LHS competencies and ability to form productive partnerships with health systems. LeaRRn offered embedded research opportunities during which rehabilitation researchers (Scholars) were paired with a health system in a hybrid/remote format to gain practical experience applying LHS principles to rehabilitation care.

## 2 Question of Interest

This manuscript focuses on the following question: What are the benefits and limitations of LeaRRn's LHS Scholar program, and what lessons can inform future training programs? We describe the structure and implementation of the program, summarize evaluation findings from the first three Scholar cohorts, and offer recommendations for strengthening future training programs.

### 2.1 The LHS Scholar Program Structure

LeaRRn was a collaboration of three universities and nine health system partners, one of which had three participating divisions. Collectively, health system partners represented geographically diverse areas of the country and provided care across the lifespan from pediatrics to geriatrics, and across settings (i.e., acute care, inpatient rehabilitation, skilled nursing, home health, and outpatient). LeaRRn's Scholar program was designed to provide rehabilitation researchers with real world experiences developing a research or quality improvement project within a health system. A central tenet of the Scholar's experience was that the project be based on, and driven by, a health system‐identified priority.

LHS Scholars were selected annually through a competitive process that invited applicants from any rehabilitation discipline at any career stage, with doctoral‐level or equivalent training/research experience. Prior to the call for applications, each health system identified a set of research or quality improvement priorities. Applicants reviewed the targeted priorities and addressed the following in the Letter of Intent: their research and clinical background, and how it aligns with one or more health system priorities; experience working with health system partners; and experience with data analysis. Top applicants were selected by LeaRRn's Executive Committee, paired with a LeaRRn faculty mentor, and matched with one or two potential health systems. Health system staff met virtually with the applicant, mentor, and LeaRRn leadership to determine whether the Scholar's background and skills were sufficiently aligned with the health system's priority. Final candidate selection was a collaborative process, with consensus among all parties to move forward.

The overall goal of the Scholar experience was to conduct the preparatory work for the research or quality improvement project. Scholar activities included: (a) refining the research or quality improvement question; (b) developing a relationship with health system stakeholders; (c) developing an understanding of the health system context; (d) identifying available data and addressing access issues; (e) developing LHS research competencies; and (f) formulating a plan/proposal with the health system to address a system‐identified priority. Ideally, at the end of the training period, the Scholar and health system would be well positioned to advance the work, for example, by seeking pilot funding through LeaRRn or other mechanisms.

The Scholar program was largely remote with funding for occasional in‐person travel to the health system site. The remote design made it a more feasible option for many candidates, who were not expected to relocate or to be already employed by a partnered health system. Scholars received modest funding to support their work and committed at least 10% of their time and effort over the course of at least 1 year. Faculty mentors also received a $1500 stipend to support the Scholar by ensuring the project maintained appropriate scope and momentum, offering methodological advice, and helping navigate challenges as they arose.

## 3 Methods

This program evaluation utilized a convergent mixed‐methods approach wherein quantitative and qualitative data were collected in parallel using the same survey instruments [14, 15]. Qualitative findings were used to contextualize and explain quantitative results. This program evaluation was deemed nonhuman subjects research by the University of Pittsburgh IRB.

For each of the three cohorts, evaluation data were primarily collected electronically from the Scholars, their mentors, and their health system partners. Data were collected using seven assessment tools, with the first survey completed approximately 6 months into the scholar year and up to 3 years after completion of their scholar activities (see Table 2). Of the seven survey tools, six were completed via Qualtrics, and one was completed collaboratively by each Scholar and mentor using a Word document. Surveys contained closed‐ended questions with Likert response options followed by an open‐ended response option in which respondents provided their perspectives and elaborated on closed item responses (Appendix B).

Two researchers (M.J.W., L.R.) with expertise conducting qualitative and mixed methods research in rehabilitation, health services, and anthropology conducted the thematic analysis. They discussed and refined code definitions. Once categories were refined, the data were reviewed again and triangulated across surveys to interpret patterns and develop preliminary themes. Themes were iteratively refined through discussion, then used in conjunction with quantitative findings to inform recommendations for future LHS research training programs. These recommendations were reviewed by the remaining members of the team who provided feedback to inform the final recommendations.

## 4 Results

Within its first 3 years, LeaRRn supported 18 LHS Scholars, most of whom were Assistant Professors and had clinical backgrounds in physical therapy (Table 1). While the Scholar program was intended to last 1 year, most projects lasted one and a half to 2 years. Below we summarize quantitative findings, then present qualitative themes that contextualize barriers, facilitators, and program impact. Finally, we present the recommendations informed by the program evaluation data.

**TABLE 1:** —LeaRRn LHS scholars years 1–3.

Scholar clinical background	Health system	Priority topic	Cohort
Physical therapist	UPMC‐RI	Transitions of care following stroke	Year 1 cohort
Physical therapist	Cleveland Clinic	Value of physical therapy in the acute care setting	Year 1 cohort
Physical therapist	UPMC‐RI	Data‐driven decision‐making for patients with low back pain	Year 1 cohort
Occupational therapist	Intermountain Health	Enhancing rehabilitation care delivery in the home	Year 1 cohort
Physical therapist	AHCA/NCAL	Analyzing changes in functional outcomes in older adults in skilled nursing facilities	Year 2 cohort
Physical therapist	Johns Hopkins Medicine	Impact of an activity and mobility promotion program on cognition	Year 2 cohort
Physical therapist	Intermountain Health	Treatment of knee osteoarthritis via telehealth	Year 2 cohort
Physical therapist	Boston Medical Center	Measuring patient mobility across the rehabilitation continuum of care	Year 2 cohort
Occupational therapist	Cleveland Clinic	Value of occupational therapy in the acute care setting	Year 2 cohort
Physical therapist	UPMC‐RI	Predicting outcomes of total shoulder arthroplasty	Year 2 cohort
Physical therapist	Boston Medical Center	Implementing an activity and mobility promotion program	Year 2 cohort
Physical therapist	Cleveland Clinic	Interpreting common measures of function in outpatients with neurologic conditions	Year 3 cohort
Speech‐language pathologist	Intermountain Health	Identifying falls risk in patients in need of speech‐language pathology services	Year 3 cohort
Occupational therapist	UPMC‐RI	Examining the impact of cognitive deficits on health system outcomes	Year 3 cohort
Occupational therapist	UPMC‐CAT	The impact of mobility device use on reduced falls and healthcare costs	Year 3 cohort
Physical therapist	BAYADA Home Health	CMS policy and payer impacts on home health utilization and outcomes	Year 3 cohort
Speech‐language pathologist	Johns Hopkins Medicine	The prognostic value of cognitive metrics on health outcomes	Year 3 cohort
Occupational therapist	UPMC‐IHS	Assessing the quality of an advanced illness care program	Year 3 cohort

Abbreviations: AHCA/NCAL, American Health Care Association/National Center for Assisted Living; UPMC, University of Pittsburgh Medical Center; UPMC‐RI, UPMC Rehabilitation Institute; UPMC‐CAT, UPMC Center for Assistive Technology; UPMC‐IHS, UPMC Innovative Homecare Solutions.

### 4.1 Quantitative Findings

Overall response rates for the surveys ranged from 73% to 100% (Table 2).

**TABLE 2:** Evaluation tools for LHS Scholar program.

Tool	Completed by	Description	Frequency	Eligible sample	Number of responses	Average number of surveys	Overall response rate
Scholar performance assessment	Scholar and mentor	A document that outlines the scholar's goals, plans, and accomplishments with a focus on progress with the project and development of LHS competencies.	Completed every 6 months. Each scholar and mentor pair (*n* = 18) was eligible to complete this assessment at least three times.	56	51	2.8×/per scholar	91%
Health system assessment of Scholar and mentor	Health system collaborator(s)	A 10‐item survey with Likert scale and free text response options assessing the scholar's communication, engagement, and effectiveness in advancing the project; and assessing the mentor's contribution in guiding the scholar and the project.	Completed every 6 months. Each health system was eligible to complete this assessment at least three times for each scholar.	56	52	3×/per scholar	93% (for survey completion by at least one individual at each health system at each time point)
Scholar assessment of mentor	Scholar	A seven‐item survey with Likert scale and free text response options assessing the mentor's responsiveness and assistance with the scholar's project.	Completed Every 6 months. Each scholar was eligible to complete this survey at least three times.	57	55	3.1×/per scholar	96%
Mentor quarterly check‐ins with LeaRRn leadership	Mentor	An eight‐item survey with Likert scale and free text response options assessing the mentor‐mentee relationship.	Completed every 3–4 months. Each mentor was eligible to complete this survey at least three times.	63	56	3.3×/per scholar	89%
Scholar assessment of project	Scholar	A 13‐item survey with Likert scale and free text response options assessing the scholar's experience with the health system including collaborations and successes and challenges.	Completed at the end of the scholar period.	18	18	1	100%
Health system assessment of LeaRRn	Health system partners	A 13‐item survey with Likert scale and free text response options assessing LeaRRn's communication, didactic activities, website, and impact on the health system.	Completed annually by representatives from health system partners (*n* = 9).	27 (9 health systems × 3 years)	19 responses from at least one health system member over the 3 years	Two health system members per year	70% (for survey completion by at least one health system at each time point)
Post‐Scholar survey	Scholar	A five‐item survey asking the scholar about subsequent funding and work as a result of the scholar program and continued engagement with the health system.	Completed annually for 3 years following the end of the scholar period.	Eleven scholar eligible to complete the first post scholar survey. Four Scholars eligible to complete the second postscholar survey. (*n* = 15 surveys)	13	1.2× per/scholar	87%

#### 4.1.1 Health System Assessment of Scholar, Mentor, and Project

Figure 1 summarizes findings from the first and final health system surveys showing little difference in responses over time. Over 80% strongly agreed that the Scholar's work addressed a health system priority topic, and 70%–80% strongly agreed that the Scholar was working effectively with the health system and sharing ideas and expertise. Seventy percent strongly agreed that the mentor effectively guided the Scholar and the project. Eighty percent strongly agreed that the Scholar's work had or would lead to improvements in care.

**FIGURE 1:**
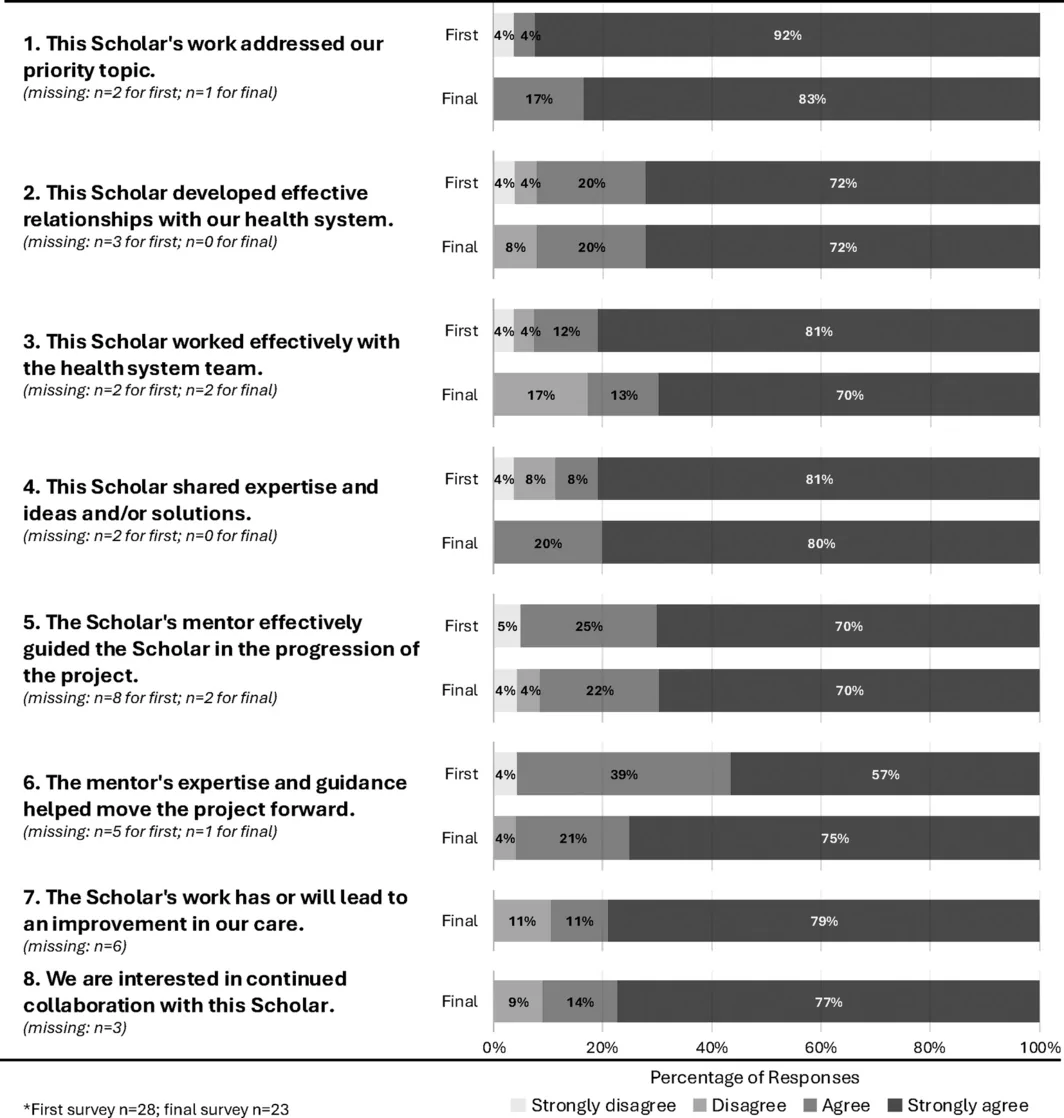
Health system assessment of scholar, mentor, and project*.

#### 4.1.2 Scholar and Mentor Assessments

Figure 2A summarizes findings from the Scholar assessment of the mentor. There were no notable differences between the first and the last survey. Approximately 60%–80% strongly agreed the mentor helped them strategize establishing relationships and refining and implementing projects. Slightly fewer strongly agreed the mentor helped with data access, use, and analysis. Figure 2B summarizes findings from the first and last mentor survey for each Scholar. A greater proportion of mentors strongly agreed they were contributing to the Scholar's development at the final survey (70%) relative to the first survey (45%). Almost 80% of mentors strongly agreed they were regularly engaged with the Scholar throughout the period. Less than 50% strongly agreed the plans for the year were reasonable, but none disagreed with this statement.

**FIGURE 2:**
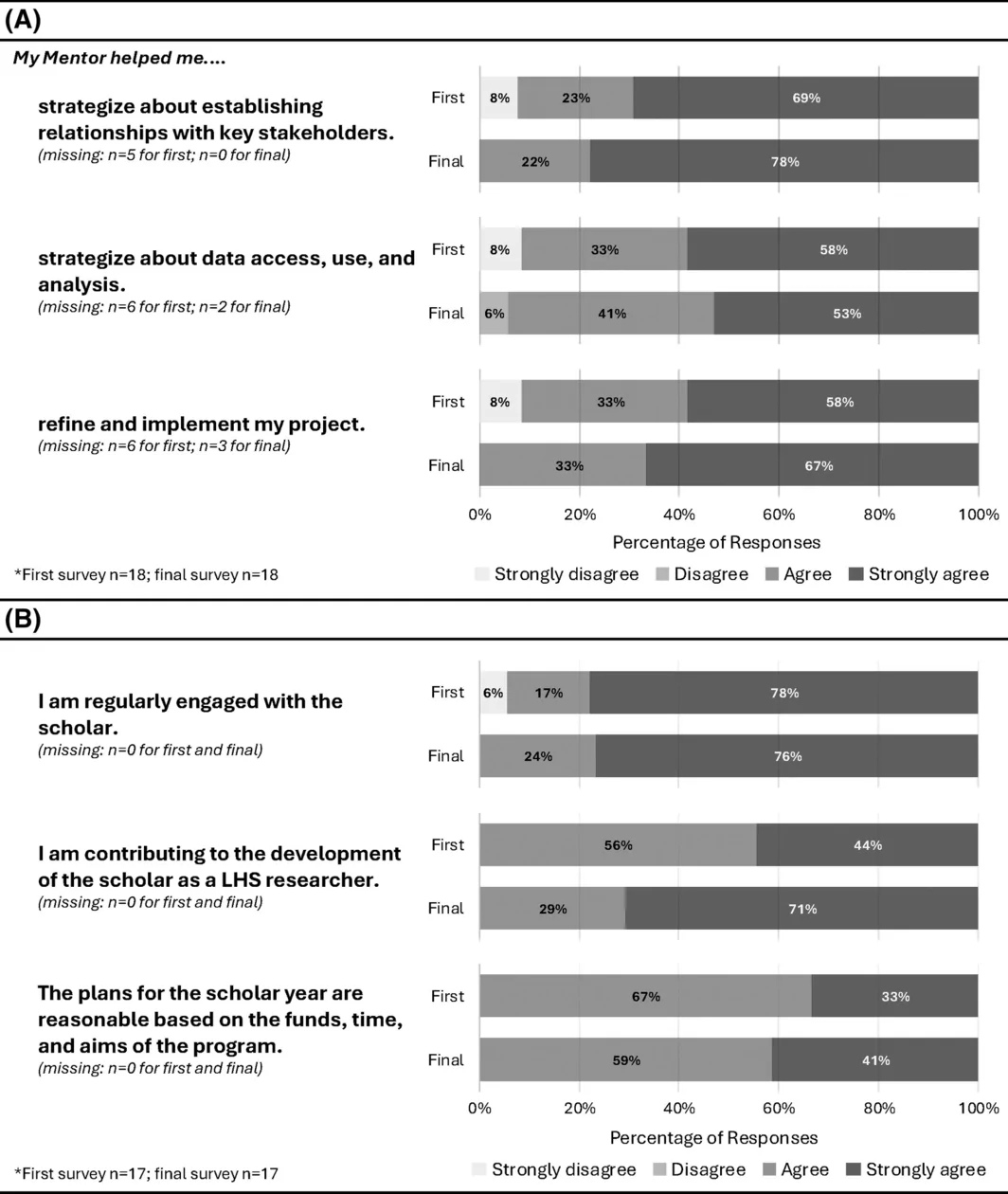
Scholar and mentor assessments*. (A) Scholar assessment of mentor. (B) Mentor quarterly check‐ins.

#### 4.1.3 Assessments of Scholar Experience

Scholars reported they collaborated with a variety of stakeholders (Table 3). Clinicians and administrators were the most frequent responses, and payers and suppliers were the least frequent. Figure 3 summarizes the facilitators and barriers to project success. A majority of Scholars indicated leadership support, their clinical knowledge, and alignment with health system priorities were facilitators for project success. Information technology and alignment with health system priorities were the most frequent barriers noted by a third of the Scholars.

**TABLE 3:** Collaborators on the Scholar projects (*n* = 18).

Which of the following groups did you collaborate with during your scholar project?	*n*	%
Administration	14	77.8
Clinicians	15	83.3
External care providers	6	33.3
Informatics	12	66.7
Patients	4	22.2
Payers	3	16.7
Quality improvement specialist	6	33.3
Suppliers	4	22.2

**FIGURE 3:**
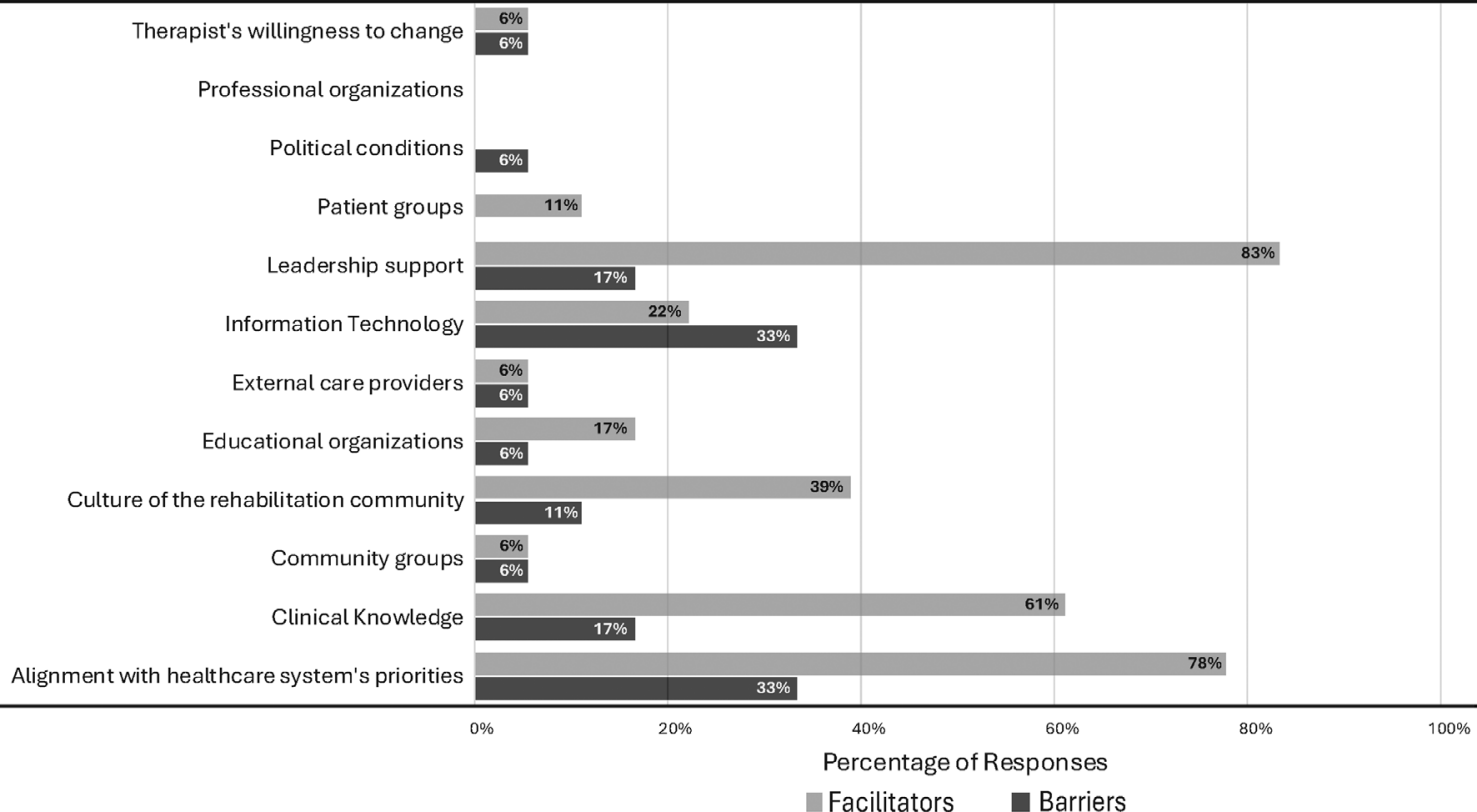
Facilitators and barriers for project success (*n* = 18).

Fifty percent of the Scholars reported they had advanced their skills in all 7 LHS competency areas originally defined by AHRQ [15] during the training period. The remainder reported working on 5–6 of the 7 competencies. The majority of Scholars reported skill advancement in Engagement, Leadership, and Research Management (89%); Research Questions and Standards of Scientific Evidence (83%); Research Methods (83%); Systems Science (61%); Ethics of Research and Implementation in Health Systems (61%); and Informatics (56%). Skill advancement was lowest in Improvement and Implementation Science (33%).

#### 4.1.4 Health System Assessment of LeaRRn

When asked about the degree of the health system's involvement with LeaRRn, 78.8% indicated they felt the amount of involvement was “just right” and 18.2% indicated it was “too little.” A majority (97.1%) agreed or strongly agreed that their involvement with LeaRRn had motivated their health system to develop as a learning rehabilitation health system. Figure 4 summarizes the impact of LeaRRn from the health system perspective. A majority indicated that quality improvement and staff use of evidence‐based practice improved moderately or significantly. A majority also indicated they had minimal or no improvement in cost savings, staff involvement with continuing education, professional advancement, and effectiveness of care. These domains were not explicit targets of the Scholar projects and likely require more time and broader organizational change to demonstrate measurable impact.

**FIGURE 4:**
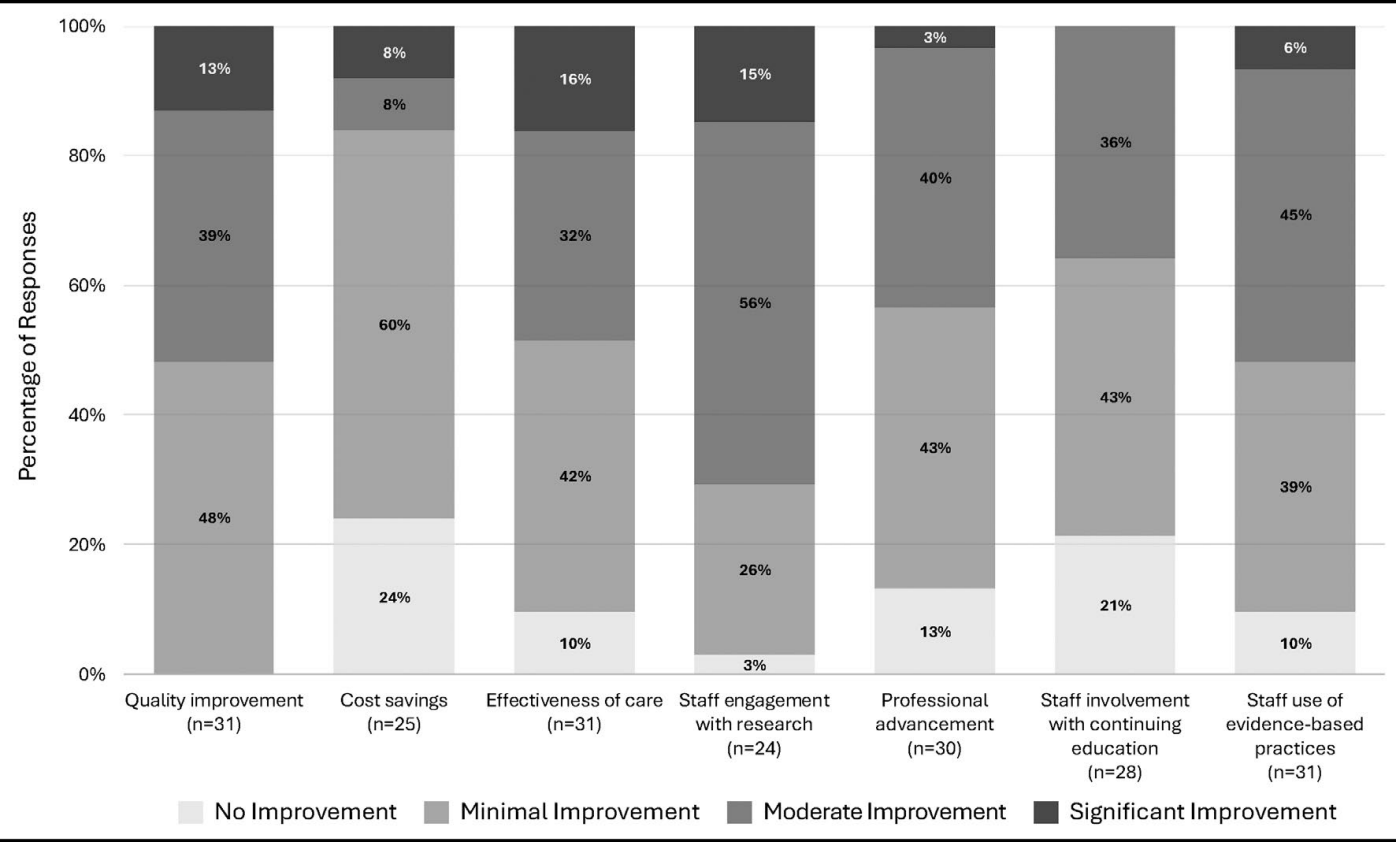
Health system assessment of LeaRRn.

#### 4.1.5 Postscholar Surveys

Over half of the responding Scholars indicated they had submitted a grant proposal; three had received external funding (2 NIH‐funded pilot projects through LeaRRn and 1 had received a faculty fellowship through foundation funding) (Table 4). Fifty percent reported continuing quality improvement work with their LeaRRn health system partner and/or another health system. Sixty percent reported continued work with their mentor and 70% reported continued engagement with their health system partner and other LHS Scholars. Finally, 80% strongly agreed and 20% agreed that LeaRRn continued to impact their work.

**TABLE 4:** Postscholar work developing from the LHS Scholar project (*n* = 10).

Activity	*N* (%)
Grant proposal submissions	6 (60)
Externally funded projects	3 (30)
Internally funded projects	0
Quality improvement work with a health system	5 (50)
Publications	5 (50)
Conference presentations	7 (70)

### 4.2 Qualitative Findings

The final coding categories, their definitions, and associated themes are shown in Table 5. In the text below we expand on the eight themes related to barriers and challenges (B), facilitators (F), and successes and impacts (S). Rich‐text exemplars associated with these themes are shown in Appendix A.

**TABLE 5:** Coding and thematic analysis.

Final codes and definitions				
Mentor role (M)	Health system priorities, engagement and alignment (H)	Barriers to Scholar work (B)	Facilitators of Scholar work (F)	Successes and impact (S)
The mentor‐mentee relationship, mentor expertise, type and frequency of meetings, amount of engagement in health system activities, role and functions of the mentor, two and impact on Scholars.	The development of the health system's rehabilitation priorities, and their alignment with broader health system and Scholar interests; alignment of health system and scholar expectations and goals; match of Scholar skills and expertise with project needs, the stakeholders engaged, the health system's support of the work, and the process of project identification and co‐design.	Factors identified by Scholars, health systems and/or mentors that were barriers to the Scholar's work within the health system.	Factors identified by Scholars, health systems and/or mentors that facilitated the Scholar's work within the health system.	Products and collaborations resulting from the Scholar work; how the Scholar program impacted the Scholar's LHS research competencies, skill development and career trajectory; and the program's impact on health system operations and capacity.
Themes				
M1: Regular meetings with academic mentors helped Scholars focus ideas and resolve challenges.	H1: Most Scholars worked with a team of stakeholders at the health system, including administrators, clinicians, external care providers, informatics, patients, and quality improvement specialists.	B1: Identifying a focused project that was feasible to initiate, execute, and complete in a 1 year period was challenging for most Scholars.	F1: Mentors who are actively involved serve as sounding boards and provide guidance that facilitates success.	S1: LHS scholar work led to peer‐reviewed manuscripts, reports, conference posters, and presentations (at conferences, health systems, LeaRRn, and/or at home institutions).
M2: Mentors help Scholars access resources for skill development, funding opportunities, analyses, and dissemination.	H2: Scholars and health systems worked together to co‐design Scholar projects that aligned with priorities established by the health system.	B2: Competing professional demands and limited protected time for Scholar activities was a barrier to health system work.	F2. Mentors whose research background and/or topic knowledge helped Scholars develop their project, engage with the health system, or advance other related goals.	S2: Scholars provided many examples of the ways in which they had advanced their knowledge and skills in LHS Research competencies and described a range of learning activities in addition to their research experience that supported their growth.
M3: Mentors had varying levels of involvement with the health system.	H3: Some health systems noted that only a few staff engaged with LeaRRn Scholars, which limits the impact of the program.	B3: Problems with timeliness, responsiveness and/or perceived lack of engagement of either the Scholar or health system partners disrupted steady progress in some cases.	F3: Academic mentors who were affiliated with or familiar with the health system and LHS research were in a unique position to help access data, and navigate health system relationships and resources.	S3: Some Scholars commented on how the skills that they acquired during the Scholar program would likely be applied to other work.
M4: Mentors helped Scholars navigate data access and IRB processes, recognize the expertise they needed, and in some cases identify appropriate variables in the EMR.	H4: The priorities of the team or department do not necessarily align with the priorities of the overall health system, though some health systems noted that identifying department or team priorities was easy.	B4: Staff turnover, competing clinical and administrative demands, institutional reorganization, and re‐prioritization within health systems creates challenges for continuity of the project, engagement, and communication.	F4: Strong health system collaboration and engagement facilitates data access and development of impactful projects.	S4: Some Scholars continued to work with their LeaRRn mentors even after the formal Scholar work ended.
	H5: Health system priorities may shift over time due to external considerations, institutional priorities, or leadership changes.	B5: Regulatory requirements, including the need to initiate data use agreements, navigate regulatory requirements and undergo one or more rounds of IRB review, often took many months which delayed or impeded access to data.	F5: Scholars who are already health system employees have a clear advantage in understanding the health system context and operations.	S5: Scholar projects can have great value and positive impact within a health system.
	H6: Many Scholars were interested and motivated to pursue funding opportunities to extend the collaboration, while health systems varied in their interest, motivation, and capacity.	B6: Project timelines were impacted by the complexity of using multiple sources of health system data, data availability, and/or need to access limited or busy IT expertise to assist with data acquisition and analysis.	F6: Strong research skills that aligned with the Scholar project needs, and strong alignment of priority interests, facilitated successful completion of work.	S6: Scholars can provide valuable assets and expertise to strengthen health system capacity.
		B7: Scholars sometimes needed to pivot their initial plans to overcome challenges or address shifting health system priorities.	F7: Scholar's strong communication, time management, organizational and interpersonal skills, self‐direction, flexibility to pivot as needed, and tactfulness facilitated successful engagement with the health system, and project success.	S7: Development of strong partnerships with health systems set the stage for continued work and collaboration on grant proposals.
		B8: Suboptimal health system‐scholar matches due to misaligned interests, lack of appropriate research skills, and/or content knowledge created tensions between the Scholar and health system.	F8: Scholars benefit from sharing knowledge from other Scholars within and across LeaRRn Scholar cohorts.	S8: Partnered research strengthens the health system's capacity for future LHS work.

#### 4.2.1 Barriers to Scholar Work

B1: *Identifying a focused project that was feasible to initiate, execute, and complete in a 1 year period*. The 12‐month program was considered too brief by many Scholars, and some commented they were unable to complete their proposed work. In some cases, it took a Scholar months to narrow down a research question and develop a plan of activities. Several Scholars reported extending their health system engagement beyond the 1 year period “with no funding or resources.”

B2: *Competing professional demands and limited protected time for Scholar activities*. Some Scholars commented that funding provided by LeaRRn was very modest, resulting in limited protected time and challenges balancing the health system work with competing professional demands.

B3: *Problems with timeliness, responsiveness, and/or perceived lack of engagement of either the Scholar or health system partners*. Some Scholars and health system stakeholders commented that steady progress was hampered because of the availability of either the Scholar or health system collaborator due to, for example, leaves of absence or changing job responsibilities.

B4: *Staff turnover, competing clinical and administrative demands, institutional reorganization, and re‐prioritization within health systems*. Engagement with health system staff was sometimes challenging due to competing productivity and scheduling demands. Some clinicians had to meet outside of regular work hours due to the lack of financial support for their program participation. Some Scholars reported challenges with project continuity when there was staff turnover, institutional reorganization, and re‐prioritization within the health system.

B5: *Regulatory requirements, including the need to initiate data use agreements*. Regulatory issues often took many months, which delayed or impeded access to data. Most Scholar projects were considered quality improvement and exempt from IRB review, but health systems were not familiar with collaborative projects involving external scientists, and internal reviews and checks were often required. Given the brevity of the training program, some Scholars were not able to secure data in a timely manner and/or complete data analyses.

B6: *Project timelines were impacted by the complexity of using multiple sources of health system data, data availability, and/or the need to access limited or busy IT expertise*. Scholars who used health system data reported on its complexity, the challenges of linking data from multiple sources, the lack of availability of particular data elements, and/or the need for help from health system IT staff who had limited time.

B7: *Scholars sometimes needed to pivot their initial plans to overcome challenges or address shifting health system priorities*. This was most evident for Cohort 1 who entered the program in 2020 and found that the Covid‐19 pandemic had “a drastic effect on the immediate priorities of the LHS.”

B8: *Suboptimal health system matches due to misaligned interests, lack of appropriate research skills, and/or lack of content knowledge*. Scholars who did not embrace the health system priority had weaker health system partnerships. Scholars who lacked appropriate skills and knowledge required substantial methodologic support from mentors and health system partners to progress the project.

#### 4.2.2 Facilitators of the Scholar Program

F1: *Actively involved mentors served as sounding boards and provided guidance that facilitated success*. Successful LeaRRn Scholars met regularly with their mentors throughout the training and mentors were available as needed to discuss their work.

F2: *Mentors whose research background and/or content knowledge aligned with the health system project helped Scholars develop their project, engage with the health system, or advance other related goals*. Such background/knowledge helped mentors to recognize Scholars' needs and provide guidance or referral for technical/methodologic expertise.

F3: *Mentors who were affiliated with or familiar with the health system and LHS research were able to help Scholars access data and navigate health system relationships and resources*. LeaRRn mentors who were familiar with the health system and LHS research, through working with prior trainees or projects or because of institutional affiliations, were in a unique position to help “cut red tape,” to extract data more efficiently.

F4: *Strong health system collaboration and engagement facilitates data access and development of impactful projects*. Strong health system relationships facilitated deeper Scholar understanding of the health system context, and set the stage for continuing or additional collaboration. Active engagement with health system team members, either in person or virtually, were key to a successful experience. Such engagement often involved multidisciplinary stakeholders.

F5: *Scholars who are already health system employees have a clear advantage in understanding the health system context and operations*. Such Scholars have established relationships within the health system and easier access to resources.

F6: *Strong research skills that aligned with the project needs, and strong alignment of priority interests facilitated successful completion of work*. Academic and research skills, such as knowing how to conduct a literature review to assess available evidence, understanding how to formulate research questions, and the ability to design a research or quality improvement project, helped Scholars succeed.

F7: *Scholar's strong communication, time management, organizational and interpersonal skills, self‐direction, flexibility to pivot as needed, and tactfulness facilitated successful engagement with the health system and project success*. These personal and professional characteristics and qualifications were key to a successful experience. These and other traits, such as conscientiousness, were valued by health systems and credited with facilitating success.

F8: *Scholars benefit from sharing knowledge within and across LeaRRn cohorts*. Scholars commented that they benefited from the quarterly Scholar meetings convened by LeaRRn and through more informal networking, reporting that these interactions helped them navigate issues such as regulatory issues, data use agreements, or contextual factors.

#### 4.2.3 Program Successes and Impact

S1: *Scholar work led to peer‐reviewed manuscripts, reports, conference posters, and presentations (at conferences, health systems, LeaRRn, and/or at home institutions)*. These types of products helped build Scholars' academic resumes.

S2: *Scholars provided many examples of the ways in which they had advanced their knowledge and skills in LHS Research competencies and described a range of learning activities in addition to their research experience that supported their growth*. Though each Scholar's learning activities were customized to their needs, they reported a range of activities which included short courses, workshops, conferences, independent study, and discussions with their mentors.

S3: *Some Scholars commented on how the skills they acquired during the program would likely be applied to other work*. Scholars spoke about acquiring new skills, for example, related to stakeholder engagement, and a deeper understanding of how to craft proposals to support quality improvement or implementation work.

S4: *Some Scholars continued to work with their LeaRRn mentors even after the formal work ended*. In some instances, mentors were engaged as co‐authors of peer‐reviewed manuscripts, newly funded pilot studies, and/or grant proposals related to the Scholar work.

S5: *Scholar projects can have great value and positive impact within a health system*. For example, Scholars noted that their work helped identify gaps in assessment and documentation practices and/or other health care improvement needs and led to actual or planned process improvements or other important contributions to their health systems.

S6: *Scholars can provide valuable assets and expertise to strengthen health system capacity*. Health system partners noted that the Scholar's energy and expertise enhanced their health system's operations and capacity for LHS activities to address priority topics.

S7: *Development of strong partnerships with health systems set the stage for continued work and collaboration on grant proposals*. The Scholar experience served as a launching pad for many trainees who reported continuing collaborative activities with their mentors and/or health system partners. As a result, some submitted and received additional funds through internal funding mechanisms, fellowships, LeaRRn pilot awards, private foundations, or federal sources to advance or continue their work.

S8: *Partnered research strengthens the health system's capacity for future LHS work*. While the nature of each Scholar's work varied, some health systems commented that the Scholar's project had enhanced their health system's capacity for future LHS work, particularly focused on rehabilitation.

### 4.3 Recommendations

The following recommendations reflect key lessons from the program evaluation. Taken together, these recommendations emphasize the importance of adequate time, aligned partnerships, active mentoring, and institutional support.

#### 4.3.1 Program Length, Support, and Structure

We recommend a minimum of 18 months for trainee projects, with flexibility to account for health system readiness, type of project, and regulatory and data access issues. Remote training programs should provide travel support for periodic in‐person health system visits to strengthen relationships and contextual awareness, and accelerate data access. Didactic education should supplement the experiential work.

#### 4.3.2 Selecting Applicants

Programs should develop a thoughtful matching process to leverage the trainee's research and clinical expertise with the health systems' needs. Training programs should be open to applicants from academic and health system settings and should select applicants who are self‐directed, flexible, and have strong communication and interpersonal skills.

#### 4.3.3 Partnerships

Health systems should have clearly defined processes to facilitate data access and minimize delays. Some models are: making researchers affiliate investigators, providing laptops with secure data access, and providing limited datasets. Training programs should offer resources and guidance (e.g., templates, legal consultation, and institutional expertise) to help trainees develop data use or other agreements.

#### 4.3.4 Mentoring

Embedded trainees should be assigned academic mentors with expertise in the content or methods needed for their project. Mentors should meet with trainees at least monthly and join health system meetings as needed. When available, mentors with knowledge of health system resources and regulatory and data access issues are especially valuable.

#### 4.3.5 Program Expectations

Trainees, mentors, and health system partners must align expectations, co‐design projects with an appropriate scope, and appreciate timelines. Program leadership should provide clear expectations for project timelines, possible variation, and the preliminary role projects may represent in the larger context of addressing a system priority.

#### 4.3.6 Health System Navigation

Health systems should provide and support a primary institutional contact to help trainees navigate the health system and facilitate health system team engagement. Trainees should recognize the importance of establishing relationships and understanding context.

#### 4.3.7 Supporting Health System Participation

Program leadership should maintain strong relationships with health system partners. Programs should budget, when possible, for health system personnel participation, technical or analytic support, and project implementation.

#### 4.3.8 Administrative Program Support

Programs should offer trainees project management support to assist with scheduling large or recurring meetings with multiple stakeholders.

#### 4.3.9 Provide Opportunities for Scholars to Learn From Each Other

Program should support peer learning through facilitated works in progress meetings with current and past trainees.

## 5 Discussion

The LeaRRn LHS Scholar program was distinctive in its focus on rehabilitation, remote format, and inclusion of diverse stakeholder perspectives in evaluation. While multiple evaluations of LHS research training programs have been published, the nature of the programs themselves, as well as the size, scope, depth, and foci of the evaluations have varied considerably [7, 8, 9, 10, 11, 12, 13, 16, 17, 18]. LeaRRn's effort requirement (10%) over 1–2 years was significantly lower than the 70%–100% effort and 2 year timeframe of some other programs [7, 10, 12]. LeaRRn trainees, who were Assistant Professors in academic settings, were most similar to those reported in the AHRQ K‐12 programs [8] and the VA's Researcher or Evaluator in Residence program [9] rather than trainees from predoctoral, postdoctoral [7], or resident programs [12].

Prior evaluations have characterized the programs themselves and provided select descriptive data on one or more trainee outcomes such as trainee satisfaction [12, 18], career outcomes and/or professional impact [9, 10]. Our evaluation employed multiple stakeholder perspectives and explored the Scholar's partnership with the health system, the mentor role, successes and challenges of the health system partnership, and the impact of the Scholar's project on the health system. Despite differences in program and evaluation format, our findings are consistent with prior program evaluations, which demonstrated gains in researcher capacity [8, 11] and LHS competencies [8, 11, 18], and led to traditional scholarly outputs [8, 9]. Barriers we identified related to data access, regulatory processes, and competing demands have been reported by others [7, 8, 9, 12].

Our recommendation for future programs emphasizes the importance of adequate time, aligned partnerships, active mentoring, and institutional support for successful embedded learning health system training. We acknowledge that the novel elements of our program, rehabilitation focus and remote format, might limit generalizability to other disciplines and fully in‐person programs. Despite this and our small sample size, we believe that our recommendations, grounded in rigorous mixed methods evaluation data, have applicability to other training programs.

Our recommendation for longer training programs parallels Vitous et al.' finding that many projects were not feasible within 1 year [9]. Our recommendation to offer peer learning opportunities aligns with Lozano et al. and McMahon et al.'s reports on the valuable experiences of scholar community‐building and networking, and the importance of facilitating a “cohort effect” [8, 13]. Our recommendations addressing ways to enhance the embedded scientist and health system relationship are consistent with practices and observations noted in other program evaluations. For example, Towfighi et al. offer consultation to help researchers align their projects with the system's business model and infrastructure [16]. Marshall et al. described the importance of providing specific training to help researchers effectively balance the perspectives of their academic and health system mentors [11], and Lozano et al. noted that regular meetings between embedded scientists and health system leadership were deemed essential to legitimize the embedded researchers presence within the system [8].

## 6 Conclusion

This evaluation provides a unique example that demonstrates the success of a remote embedded research training program for rehabilitation researchers. The program had a clear positive impact for trainees and their affiliated health systems and demonstrates the promise of this type of training model for other LHS training programs. This program evaluation yielded recommendations to strengthen future training programs related to: program length, support, and structure; applicant selection; health system partnerships; mentoring; training expectations; health system navigation; health system financial support; administrative program support; and peer‐learning opportunities.

## Funding

This work was supported by the Eunice Kennedy Shriver National Institute of Child Health and Human Development (1P2CHD101895‐01) and the National Institute of Nursing Research.

## Conflicts of Interest

The authors declare no conflicts of interest.

## Data Availability

The de‐identified data is available in supplementary materials.

## Appendix A See Table A1

**TABLE A1:** Rich text exemplars.

A. Barriers This category includes factors, identified by Scholars, health systems and/or mentors that were barriers to the Scholar's work within the health system.	
B1: Identifying a focused project that was feasible to initiate, execute, and complete in a 1 year period was challenging for most Scholars.	“At times, I feel a 2 year LHS program could be a good option to allow for a year of relationship building and planning/data access steps, followed by a year to execute the project.” (Cohort 3 scholar)
B2: Competing professional demands and limited protected time for Scholar activities was a barrier to health system work.	“Overall I think it is challenging to pursue a partnership with 15K funding… it would benefit to have 2–3 year funding to sustain relationships with health systems when we try to get external funding…” (Cohort 2 Scholar)
B3: Problems with timeliness, responsiveness and/or perceived lack of engagement of either the Scholar or health system partners disrupted steady progress in some cases.	“Primary challenges have been related to timelines (data access, sequential maternity leaves of both of the primary collaborators).” (Cohort 3 scholar)
B4: Staff turnover, competing clinical and administrative demands, institutional reorganization, and re‐prioritization within health systems creates challenges for continuity of the project, engagement, and communication.	“The pandemic created challenges for collaboration, specifically delays in the project due to the need for the LHS to focus on clinical care and deprioritize this project at times. In addition, travel restrictions… and concerns about exposing patients… prevented the originally planned travel to the LHS” (Cohort 1 Scholar)
B5: Regulatory requirements, including the need to initiate data use agreements.	“My project required coordination between multiple clinical settings and, by extension, multiple data stewards and regulatory officials… also the time it takes to receive approval (or even wait for communications) from each entity resulted in significant delays” (Cohort 1 scholar)
B6: Project timelines were impacted by the complexity of using multiple sources of health system data, data availability, and/or need to access limited or busy IT expertise to assist with data acquisition and analysis.	“Longer timeline for data access; inability to link data from the… patient data repository to the EMR, which impacted our ability to gather additional clinical information outside of what was already captured in the repository.” (Cohort 3 scholar)
B7: Scholars sometimes needed to pivot their initial plans to overcome challenges or address shifting health system priorities.	“The workgroup I was part of was quite big and they had many competing priorities, there were several times when we had to pivot or delay moving the project ahead… The QI proposal underwent significant changes since inception based on resources and… priorities.” (Cohort 2 scholar)
B8: Suboptimal health system‐Scholar matches due to misaligned interests, lack of appropriate research skills, and/or content knowledge created tensions between the Scholar and health system.	“She is very hard working but her research skills are junior level.” (Cohort 2 health system)

B. Facilitators This category includes factors, identified by Scholars, health systems and/or mentors that facilitated the Scholar's work within the health system.	
F1: Mentors who are actively involved serve as sounding boards and provide guidance that facilitates success.	“The regular meetings with my mentor during my scholar year was very helpful in solidifying my scholar plans and activities.” (Cohort 2 scholar)
F2. Mentors whose research background and/or topic knowledge helped Scholars develop their project, engage with the health system, or advance other related goals.	“[The mentor]'s research background has been invaluable in steering [the Scholar] in the right direction related to scope, design, and other technical research processes” (Cohort 2 health system)
F3: Mentors who were affiliated with or familiar with the health system and LHS research were able to help Scholars access data, and navigate health system relationships and resources.	“With a large health system the red tape is sometimes a challenge to cut and Janet has helped significantly in getting data extracted from the system” (Cohort 1 health system)
F4: Strong health system collaboration and engagement facilitates data access and development of impactful projects.	“The strong relationships [the Scholar] established… and deep understanding of the [health] system has allowed her to overcome challenges (e.g., staff shortages, COVID) to successfully implement the… program.” (Cohort 2 mentor)
F5: Scholars who are already health system employees have a clear advantage in understanding the health system context and operations.	“[The Scholar]'s plans are ambitious, however he has access to more baseline resources than typical for a researcher since he's already embedded into his health system.” (Cohort 2 health system)
F6: Strong research skills that aligned with the Scholar project needs, and strong alignment of priority interests, facilitated successful completion of work.	“I used my knowledge and training as a researcher to conduct the literature search and drafting QI internal proposals. I also helped with defining aims and purposes of the projects and also volunteered to brainstorm ideas based on what was known or not known… I used my training in implementation sciences to encourage use of stakeholder input…” (Cohort 2 scholar)
F7: Scholar's strong communication, time management, organizational and interpersonal skills, self‐direction, flexibility to pivot as needed, and tactfulness facilitated successful engagement with the health system, and project success.	“[The Scholar]'s success is possible due to her ability to plan in advance, anticipate deadlines, and prioritize tasks when multiple activities are occurring at one.” (Cohort 2 mentor)
F8: Scholars benefit from sharing knowledge from other Scholars within and across LeaRRn Scholar cohorts.	“Working with a researcher in the health system tremendously helped to navigate the DUA and IRB processes as well as study development.”(Cohort 2 scholar)

C. Successes and impact This category includes comments about products and collaborations resulting from the Scholar work; how the Scholar program impacted the Scholar's LHS research competencies, skill development and career trajectory; and the program's impact on health system operations and capacity.	
S1: LHS scholar work led to peer‐reviewed manuscripts, reports, conference posters, and presentations (at conferences, health systems, LeaRRn, and/or at home institutions).	“Presentations to various stakeholder groups as part of brainstorming and listen and learn sessions as well as final presentation to partners.” (Cohort 1 scholar)
S2: Scholars provided many examples of the ways in which they had advanced their knowledge and skills in LHS Research competencies and described a range of learning activities in addition to their research experience that supported their growth.	“I'm taking a course on Python Programming to improve my ability to work with and process data. It will also help me to better understand the ways in which data can be managed and to speak to programmers about what I need for a project.” (Cohort 2 scholar)
S3: Some Scholars commented on how the skills that they acquired during the Scholar program would likely be applied to other work.	“…I gained so much useful experience and knowledge about stakeholder engagement that continues to inform my research today. While I don't have specific plans to continue a collaboration with [the health system] right now based on the change in focus of the project and my current funding on other projects, the door is open for future partnerships.” (Cohort 1 scholar)
S4: Some Scholars continued to work with their LeaRRn mentors even after the formal Scholar work ended.	“We just submitted a paper for review last week and we are currently working on the LeaRRn pilot program project.” (Cohort 2 scholar)
S5: Scholar projects can have great value and positive impact within a health system.	“Virtual and telehealth work has been greatly enhanced with the work [the Scholar] has done.” (Cohort 2 health system)
S6: Scholars can provide valuable assets and expertise to strengthen health system capacity.	“[The health system] has the resources needed to become a LHS, but does not have a strong history of engaging in research. [The Scholar] was able to develop connections across [the health system]… to successfully conduct this project.” (Cohort 2 scholar)
S7: Development of strong partnerships with health systems set the stage for continued work and collaboration on grant proposals.	“We have established a mutually beneficial team that will continue to pursue grant funding and projects likely for the rest of my career.” (Cohort 3 scholar)
S8: Partnered research strengthens the health system's capacity for future LHS work.	“Most nonacademic healthcare organizations lack the internal resources to fully leverage data they are producing to create a project of this level of sophistication.” (Cohort 2 scholar)

## Appendix B LeaRRn Assessment Surveys

### Scholar Progress Assessments

#### Health System Collaborator Assessment of Scholar and Mentor

**Complete in Qualtrics every 6 months during their project**

**Table:**

**Form**: LeaRRn LHS Scholar Program Evaluation**—Completed by Health System**					
LHS scholar name:					
Health system name: Completed by:					
Date completed: Evaluation Period:					
For each item identified below, circle the number to the right that best matches your level of agreement. Response: 1 = Strongly disagree 2 = Disagree 3 = Neither agree nor disagree 4 = Agree 5 = Strongly agree					
**Statement**		**Response**			
This LHS Scholar's work is addressing our identified priority topic.	1	2	3	4	5
Comments and suggestions:					
2 This LHS Scholar is developing effective relationships within our health system.	1	2	3	4	5
Comments and suggestions:					
3 This LHS Scholar is working effectively with the health system team.	1	2	3	4	5
Comments and suggestions:					
4 This LHS Scholar is sharing expertise and ideas/solutions.	1	2	3	4	5
Comments and suggestions:					
5 The LHS Scholar is open to suggestions/constructive criticism.	1	2	3	4	5
Comments and suggestions:					
6 This LHS Scholar's mentor is effectively guiding the Scholar in the progression of the project.	1	2	3	4	5
Comments and suggestions:					
7 Please provide any additional feedback about the LHS Scholar, mentor, and/or status of the project.					
Comments and suggestions:					

### Scholar Final Assessments

#### Hub Collaborator Assessment of Scholar and Mentor

**Complete in Qualtrics** Health System Assessment of Scholar **at the end of the project**.

**Table:**

**Form**: LeaRRn LHS Scholar Program Evaluation**—Completed by Health System**					
LHS scholar name:					
Health system name: Completed by:					
Date completed: Evaluation Period:					
For each item identified below, circle the number to the right that best matches your level of agreement. Response: 1 = Strongly disagree 2 = Disagree 3 = Agree 4 = Strongly agree 5 = *N*/A (or don't know)					
**Statement**		**Response**			
This LHS Scholar's work addressed our identified priority topic.	1	2	3	4	5
Comments and suggestions:					
2 The LHS Scholar's work has or will lead to an improvement in our rehabilitation care.	1	2	3	4	5
Comments and suggestions:					
3 This LHS Scholar developed effective relationships within our health system.	1	2	3	4	5
Comments and suggestions:					
4 This LHS Scholar worked effectively with the health system team.	1	2	3	4	5
Comments and suggestions:					
5 This LHS Scholar shared expertise and ideas/solutions.	1	2	3	4	5
Comments and suggestions:					
6 The LHS Scholar was open to suggestions/constructive criticism.	1	2	3	4	5
Comments and suggestions:					
7 The LHS Scholar's mentor effectively guided the Scholar in the progression of the project.	1	2	3	4	5
Comments and suggestions:					
8 We are interested in continued collaboration with this LHS Scholar.	1	2	3	4	5
Comments and suggestions:					
9 Please provide any additional feedback about the LHS Scholar, mentor, and/or status of the project.					
Comments and suggestions:					

### Final Evaluation: LHS Project Assessment—to be Completed by the Scholar

**The following questions focus on your project design:**

1. How did you assemble a team?
2. Who are the members of your team?
3. What is going well with how the team functions?
4. Are there opportunities to improve the team's functioning?
5. Which of the following groups have you collaborated with? (select all that apply)
  - Administration
  - Clinicians
  - External care providers
  - Informatics
  - Patients
  - Payers
  - Quality Improvement Specialist
  - Suppliers
  - Other ___________
6. If yes, please describe how you collaborated.
7. What were the successes of collaboration within your project?
8. What were the challenges of collaboration within your project?
9. How did you use existing knowledge in your work?
10. How did your work develop new knowledge?

**The following questions focus on barriers and facilitators to project success:**

1. Which of the following were **barriers to the success** of your project? (select all that apply)
  - Alignment with healthcare system's priorities
  - Clinical knowledge
  - Community groups
  - Culture of the rehabilitation community
  - Educational organizations
  - External care providers
  - Information Technology
  - Leadership support
  - Patient groups
  - Political conditions
  - Professional organizations
  - Therapist's willingness to change
  - Other (please describe): ___________
2. Which of the following were **facilitators for the success** of your project? (select all that apply)
  - Alignment with healthcare system's priorities
  - Clinical knowledge
  - Community groups
  - Culture of the rehabilitation community
  - Educational organizations
  - External care providers
  - Information Technology
  - Leadership support
  - Patient groups
  - Political conditions
  - Professional organizations
  - Therapist's willingness to change
  - Other (please describe): ___________

**The following questions focus on the outcome of your project:**

1. What aspect of your project do you find most promising? (Or is going well?)
2. What aspect of your project has been most challenging? (Or could be going better?)
3. How did you share your work? (select all that apply)

**Table:**

Within LHS	Externally
Presentations	Presentations
Publications	Publications
Other ___________	Other ___________

4 Is there anything else you'd like to share about your project? (open field for comments)

### Scholar Progress Assessments

#### LeaRRn Scholar and Mentor Evaluation Form

**Performance Evaluation—Completed by Scholar and Mentor**

**Table:**

Scholar name:	
Evaluation period:	
Mentor name:	
Review completion date:	

**Part 1: Scholar—Complete and Give To Mentor To Review**

**Table:**

Goals and Objectives during this evaluation period:	
Accomplishments:	

Please indicate if you have advanced your competency in the following domains of LHS research (select all that apply):

**Table:**

Check if advanced	LHS DOMAINS
	**Systems Science:** *To understand how systems are financed and operate and how to apply systems theory to research and evidence implementation*. View competencies
	**Research Questions and Standards of Scientific Evidence:** *To ask meaningful questions relevant to health system stakeholders and evaluate the usefulness of scientific evidence and insights*. View competencies
	**Research Methods:** *To conduct research within the context of real‐world health systems using appropriate study designs and analytic methods to assess outcomes of interest to health systems stakeholders*. View competencies
	**Informatics*:*** *To know how to use information systems to conduct LHS research and improve patient and health system outcomes*. View competencies
	**Ethics of Research and Implementation in Health Systems*:*** *To ensure that research and quality improvement performed in health care settings adheres to the highest ethical and regulatory standards*. View competencies
	**Improvement and Implementation Science*:*** *To reduce avoidable variation in process and outcome and ensure the systematic uptake of research findings in a health system*. View competencies
	**Engagement, Leadership, and Research Management*:*** *To engage stakeholders in all aspects of the research process and effectively lead and manage LHS research teams and projects*. View competencies

LeaRRn program resources used to achieve competencies (select all that apply):

**Table:**

Check if used	LeaRRn program resources
	Webinars
	Grand rounds
	Other LeaRRn resources
	Mentor meetings

Plans to work on competencies:	

**Part 2: Mentor—Complete and Schedule Review With Scholar**

**Table:**

Evaluation summary:	
Strengths:	
Areas for development:	

**Review—Completed with Agreement from Mentor and Scholar**

**Table:**

Goals for next evaluation period:	

*_______________________________________*

*Scholar Signature*

*__________________________________________*

*Mentor Signature*

### Scholar Final Assessments

#### LeaRRn Scholar and Mentor Evaluation Form

**Performance Evaluation—Completed by Scholar and Mentor**

**Table:**

Scholar name:	
Evaluation period:	
Mentor name:	
Review completion date:	

**Part 1: Scholar—Complete and Give To Mentor To Review**

**Table:**

Goals and Objectives this evaluation period:	
Accomplishments:	

Please indicate if you have advanced your competency in the following domains of LHS research (select all that apply):

**Table:**

**Check if advanced**	**LHS DOMAINS**
	**Systems Science:** *To understand how systems are financed and operate and how to apply systems theory to research and evidence implementation*. View competencies
	**Research Questions and Standards of Scientific Evidence:** *To ask meaningful questions relevant to health system stakeholders and evaluate the usefulness of scientific evidence and insights*. View competencies
	**Research Methods:** *To conduct research within the context of real‐world health systems using appropriate study designs and analytic methods to assess outcomes of interest to health systems stakeholders*. View competencies
	**Informatics*:*** *To know how to use information systems to conduct LHS research and improve patient and health system outcomes*. View competencies
	**Ethics of Research and Implementation in Health Systems*:*** *To ensure that research and quality improvement performed in health care settings adheres to the highest ethical and regulatory standards*. View competencies
	**Improvement and Implementation Science*:*** *To reduce avoidable variation in process and outcome and ensure the systematic uptake of research findings in a health system*. View competencies
	**Engagement, Leadership, and Research Management*:*** *To engage stakeholders in all aspects of the research process and effectively lead and manage LHS research teams and projects*. View competencies

LeaRRn program resources used to achieve competencies (select all that apply):

**Table:**

**Check if used**	**LeaRRn program resources**
	Webinars
	Grand rounds
	Other LeaRRn resources
	Mentor meetings

**Part 2: Mentor—Complete and Schedule Review With Scholar**

**Table:**

Evaluation summary:	
Strengths:	
Areas for development:	

**Review—Completed with Agreement From Mentor and Scholar**

**Table:**

Plans to continue work in this area:	

*_________________________________________*

*Scholar Signature*

*_________________________________________*

*Mentor Signature*

### Scholar Quarterly Assessments

#### Mentor Quarterly Check In

**Complete in Qualtrics** Mentor Assessment of Scholar **quarterly**

**Table:**

**Form**: Mentor Quarterly Check In**—Completed by Mentor**					
Mentor name:					
LHS Scholar name:					
Health System:					
Date completed: Evaluation Period:					
For each item identified below, circle the number to the right that best matches your level of agreement. Response: 1 = Strongly disagree 2 = Disagree 3 = Agree 4 = Strongly agree 5 = *N*/A (or don't know)					
**Statement**		**Response**			
I am regularly engaged with the LHS Scholar (via email, meetings).	1	2	3	4	5
Comments and suggestions:					
2 The LHS Scholar and I are effectively communicating about the project.	1	2	3	4	5
Comments and suggestions:					
3 The LHS Scholar and I have a good working relationship.	1	2	3	4	5
Comments and suggestions:					
4 The LHS Scholar is seeking appropriate resources and advice when needed.	1	2	3	4	5
Comments and suggestions:					
5 The LHS Scholar's needs match my background and experience.	1	2	3	4	5
Comments and suggestions:					
6 I am contributing to the development of the scholar as a LHS researcher.	1	2	3	4	5
Comments and suggestions:					
7 The plans for the scholar year are reasonable based on the funds, time, and aims of the program.	1	2	3	4	5
Comments and suggestions:					
8 Any additional comments on the scholar, project, and/or health system?					
Comments and suggestions:					

### Scholar Progress Assessment

#### Scholar Assessment of Mentor

**Complete in Qualtrics every 6 months during their project**

**Table:**

**Form:** LeaRRn LHS Scholar **Mentor Review—Completed by LHS Scholar**					
Scholar name:					
Mentor name:					
Date completed: Evaluation Period:					
For each item identified below, circle the number to the right that best matches your level of agreement. Response: 1 = Strongly disagree 2 = Disagree 3 = Neither agree nor disagree 4 = Agree 5 = Strongly agree					
**Statement**		**Response**			
My mentor is responsive to my needs.	1	2	3	4	5
Comments and suggestions					
2 My mentor helps me strategize about establishing relationships with key health system personnel and other stakeholders.	1	2	3	4	5
Comments and suggestions					
3 My mentor helps me identify relevant training and resources.	1	2	3	4	5
Comments and suggestions					
4 My mentor is supporting the development of a funding proposal.	1	2	3	4	5
Comments and suggestions:					
5 My mentor helps me strategize about data access, use, and analysis.	1	2	3	4	5
Comments and suggestions:					
6 My mentor helps me to refine and implement my project.	1	2	3	4	5
Comments and suggestions:					
7 Do you have any additional comments to improve the Scholar/Mentor relationship?	1	2	3	4	5
Comments and suggestions:					

### Scholar Final Assessments

#### Scholar Assessment of Mentor

**Complete in Qualtrics at the end of the project**

**Table:**

**Form:** LeaRRn LHS Scholar **Mentor Review—Completed by LHS Scholar**					
Scholar name:					
Mentor name:					
Date completed: Evaluation Period:					
For each item identified below, circle the number to the right that best matches your level of agreement. Response: 1 = Strongly disagree 2 = Disagree 3 = Neither agree nor disagree 4 = Agree 5 = Strongly agree					
**Statement**		**Response**			
My mentor was responsive to my needs.	1	2	3	4	5
Comments and suggestions:					
2 My mentor helped me strategize about establishing relationships with key health system personnel and other stakeholders.	1	2	3	4	5
Comments and suggestions:					
3 My mentor helped me identify relevant training and resources.	1	2	3	4	5
Comments and suggestions:					
4 My mentor supported the development of a funding proposal.	1	2	3	4	5
Comments and suggestions:					
5 My mentor helped me strategize about data access, use, and analysis.	1	2	3	4	5
Comments and suggestions:					
6 My mentor helped me to refine and implement my project.	1	2	3	4	5
Comments and suggestions:					
7 Do you have any additional comments to improve the Scholar/Mentor relationship?	1	2	3	4	5
Comments and suggestions					
